# Investigation of the Surface of Ga–Sn–Zn Eutectic Alloy by the Characterisation of Oxide Nanofilms Obtained by the Touch-Printing Method

**DOI:** 10.3390/nano9020235

**Published:** 2019-02-09

**Authors:** Alexandra Dobosz, Torben Daeneke, Ali Zavabeti, Bao Yue Zhang, Rebecca Orrell-Trigg, Kourosh Kalantar-Zadeh, Anna Wójcik, Wojciech Maziarz, Tomasz Gancarz

**Affiliations:** 1 Institute of Metallurgy and Materials Science, Polish Academy of Sciences, 30-059 Krakow, Poland; a.wojcik@imim.pl (A.W.); w.maziarz@imim.pl (W.M.); tomasz.gancarz@imim.pl (T.G.); 2 School of Engineering, RMIT University, Melbourne, Victoria 3001, Australia; torben.daeneke@rmit.edu.au (T.D.); ali.zavabeti@rmit.edu.au (A.Z.); baoyue.zhang@student.rmit.edu.au (B.Y.Z.); s3486475@student.rmit.edu.au (R.O.-T.); k.kalantar-zadeh@unsw.edu.au (K.K.-Z.)

**Keywords:** liquid alloys, 2D materials, thin films, Ga–Sn–Zn alloys, gallium alloys, nanoanalysis

## Abstract

Ga–Sn–Zn eutectic alloy is a non-toxic liquid metal alloy which could be used in a multitude of applications, including as a heat transfer agent, in gas sensing, and in medicine. Alloys containing gallium readily oxidise in air, forming a thin oxide layer that influences the properties of liquid metals and which has not been studied. In this study, the oxide layer formed on Ga–Sn–Zn alloy was transferred at room temperature onto three substrates—quartz, glass and silicon. The contact angle between the liquid alloy and different substrates was determined. The obtained thin oxide films were characterised using atomic force microscopy, X-ray photon spectroscopy, and optical and transmission electron microscopy. The contact angle does not influence the deposition of the layers. It was determined that it is possible to obtain nanometric oxide layers of a few micrometres in size. The chemical composition was determined by XPS and EDS independently, and showed that the oxide layer contains about 90 atom % of gallium with some additions of tin and zinc. The oxides obtained from the eutectic Ga–Sn–Zn liquid alloys appear to be nanocrystalline.

## 1. Introduction

Liquid metal alloys based on gallium are a group of metallic alloys, liquid at room temperature, that could possibly replace toxic materials such as mercury or alloys based on lead. The most commonly used alloy based on gallium is the eutectic Ga–In–Sn alloy Galinstan, which has potential to be used in many fields including electrical engineering, computer engineering [1] and medicine [2,3]. Eutectic Ga–In–Sn alloy is currently being studied intensively as an effective micro-cooling agent with superior power-handling properties [4,5,6]. Other alloys have been proposed as a cheaper alternative to Galinstan, based on gallium, tin and zinc. These include Ga–Sn–Zn eutectic alloy [7]. Liquid metal alloys have a range of advantages and could find uses in many different applications. Those alloys exhibit excellent thermal and electrical conductivity, are safe to handle due to almost no vapour pressure [8], and exhibit a high supercooling effect [7]. Moreover, the surface of liquid gallium and its alloys oxidises easily, forming an oxide skin on the alloy [9,10,11]. The oxide skin on pure gallium has been investigated [12,13], but there are only limited data on oxides forming on alloys containing gallium as the main component. In the case of pure gallium, according to reference [12], the thickness of the solid oxide film is about 0.5 nm. For ambient oxygen pressure and temperatures, it was proposed that isotropic diffusion aggregation was the process by which the gallium oxide grows and expands on the surface of liquid gallium [13]. The oxide first grows in a fractal-like pattern, then interfacial smoothening occurs, which may be caused by insoluble impurities in the oxide [13].

This study focuses on gallium-based alloys with tin and zinc additions. The Ga–Sn–Zn ternary system is formed from three simple eutectic systems [14,15,16]. The Ga–Sn–Zn eutectic alloy, with a composition of 90.15 of Ga, 6.64 of Sn, and 3.21 of Zn (atom %) corresponding to 86.3, 10.8, and 2.9 (wt %), has a melting point of 288 K and a solidification point of 266.5 K [7]. It has been found that adding a fourth element to the ternary eutectic system can increase the difference between the melting and solidification temperature [17,18]. By adding 0.5 atom % aluminum to Ga–Sn–Zn, the difference between the two temperatures increases from 20.6 K to 65.8 K, with a melting point of 301.3 K and a solidification point of 235.5 [17]. Similarly, indium additions cause the solidification temperature to drop even further to 231 K in the case of 14.7 atom % In, with a low melting point of 289 K [18].

As gallium alloys remain liquid over a large temperature range, touch-printing can be used in order to obtain nanometric films from the surface of the alloys [19]. The method developed in reference [19] allows the transfer of oxidised material on the liquid metal surface to virtually any substrate. The method has been used to obtain 2D Ga_2_O_3_, HfO_2_, Gd_2_O_3_, and Al_2_O_3_ [19]. Due to the low costs of fabrication of these nanometric films, the materials could be used in many important fields of science and industry, including as ultra-thin insulator dielectrics in field-effect transistors, for energy storage, and in gas sensing [20]. Using gallium alloys with additions of different elements could lower the synthesis temperature of chosen oxides; however, it is crucial to understand how those additions will affect the final composition of the oxide layer and which elements will be incorporated into the obtained material. In this work, we aimed to obtain 2D layers using the touch-printing technique on silicon, glass and quartz, in order to characterise the oxide layer formed on the surface of liquid eutectic Ga–Sn–Zn alloy using atomic force microscopy, X-ray photon spectroscopy, and optical and transmission electron microscopy.

## 2. Experimental

The Ga–Sn–Zn eutectic alloy was prepared in IMMS PAS using pure Ga (99.99999% PPM Pure Metals), Sn and Zn (99.999% Alfa Aeaser) in a glovebox under a protective atmosphere of high-purity argon (99.9999% Air Products). The oxygen, nitrogen and water vapour concentrations were lower than 0.1 ppm in order to avoid the oxidation of the prepared liquid alloys. The elements were melted together and homogenised in a graphite crucible in an electrical furnace placed in the glovebox.

The thin films were obtained at room temperature in ambient air by placing a drop of liquid metal on a glass substrate. In the next step, the drop was touched with the chosen substrate. The oxide skin attached itself onto the substrate with some residual liquid metal. The liquid metal was then cleaned off the substrate by dipping the material in boiling water and wiping it with Kimwipes. The material thus cleaned and dried was analysed. Various substrates were tested, including glass, quartz and silicon. The substrates were cleaned twice before the process, in isopropanol, ethanol and water. 

In order to determine the substrate on which the deposition would be easiest, and, consequently, on which the best quality of films could be obtained, the wettability of the Ga–Sn–Zn eutectic alloy on different substrates was measured at room temperature in ambient air, outside the glovebox, in order to imitate the same conditions in which the layers are deposited on the substrates. The contact angles were measured using the static sessile drop method in IMMS PAS.

Moreover, the obtained thin films were studied at RMIT University by atomic force microscopy (Dimension Icon-Bruker with scan-assist software), and the thickness of the layers was determined. Due to the thickness of the layers, X-ray photoelectron spectroscopy (Thermo Scientific K Alpha XPS) was used to analyse the chemical composition, as this is a suitable method in the case of nanometric materials. The X-ray source was a monochromated Al Kα source. The samples on 3 mm TEM copper grids were also studied by transmission electron microscopy using a Tecnai G2 F20 operating at 200 keV in IMMS PAS. The chemical composition was also assessed using an energy-dispersive X-ray (EDX) microanalyser in TEM.

## 3. Results and discussion

**Figure 1:**
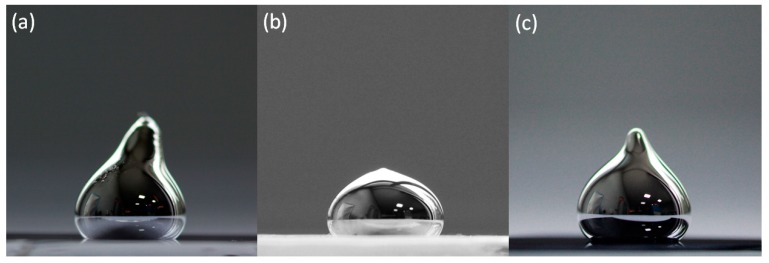
Representative contact angles for (**a**) glass, (**b**) quartz and (**c**) silicon.

The contact angle between the Ga–Sn–Zn eutectic and the substrates, namely, silicon, glass and quartz, was analysed first. For each material, the contact angles were measured three times. Representative images of the contact angles are presented in Figure 1.

The obtained contact angles were as follows: 132 ± 1° for glass, 118 ± 2° for quartz and 134 ± 1° in the case of silicon. From the conducted measurements, it can be concluded that the wetting behaviour of Ga–Sn–Zn eutectic alloy is similar in the cases of glass and silicon. The obtained values in the case of silicon are similar to the value obtained in reference [21] for Galinstan on silicon (139° at 303 K). However, it was found in reference [22] that Galinstan was found to be non-wetting on different substrates, including glass, when not oxidised. Furthermore, it was determined that the moment the oxide skin forms on the surface of Galinstan, the alloy sticks on surfaces and gives the illusion that Galinstan wets the substrates [22].

**Figure 2:**
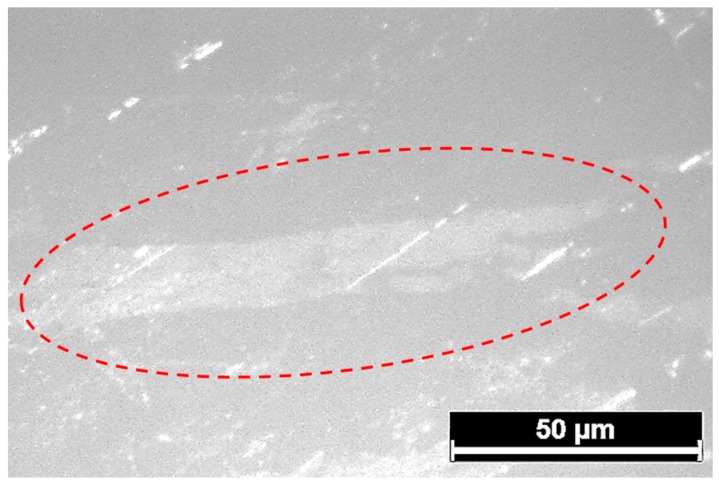
Optical microphotograph of thin film obtained from Ga–Sn–Zn eutectic alloy on glass (the layer has been circled).

In relation to 2D materials, our investigations are focused on obtained layers which were analysed using optical microscopy, scanning electron microscopy, atomic force microscopy and X-ray photoelectron spectroscopy. An optical micrograph of the nanolayers obtained on silicon is presented in Figure 2. 

**Figure 3:**
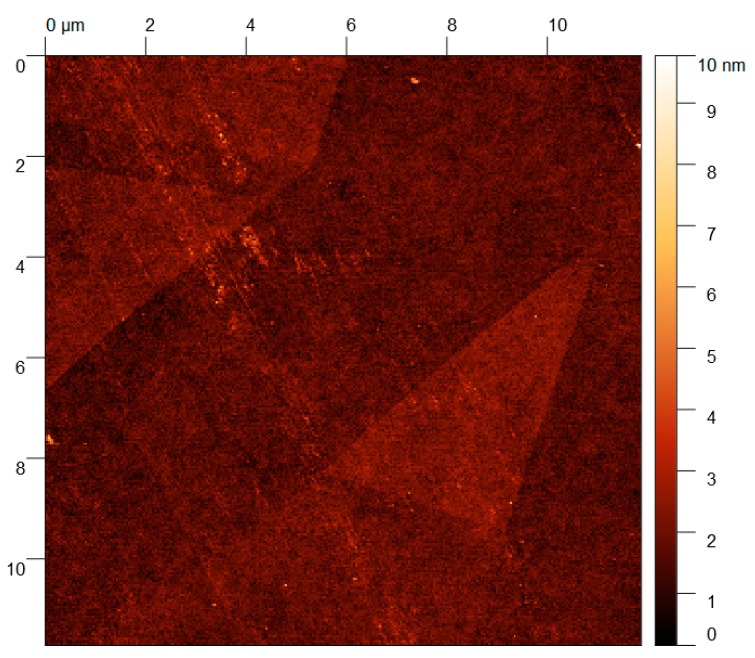
Image from AFM of nanolayers obtained from Ga–Sn–Zn eutectic alloy on silicon.

As can be seen from Figure 2, the oxide layer obtained by the touch-printing method is continuous, although there are some traces of metal left on the surface of the sample. The same microstructure is observed in the cases of all different substrates. In the next step, the layers were further analysed using atomic force microscopy. For example, the layers obtained from the Ga–Sn–Zn eutectic alloy on the silicon substrate are presented in Figure 3. 

Using AFM, the layer appears to be continuous. The measured thickness of the layers obtained from Ga–Sn–Zn eutectic alloy is about 1.5 nm. Due to the low thickness of the sample, comparable to background noise, the line depth profile is not shown. The layers obtained on glass and quartz were of similar thicknesses, ranging from 1.5 to 3 nm in the thinnest parts. The thickness of the thin films is 3 times the thickness of the layer of the oxide skin on pure liquid gallium reported in reference [12].

**Figure 4:**
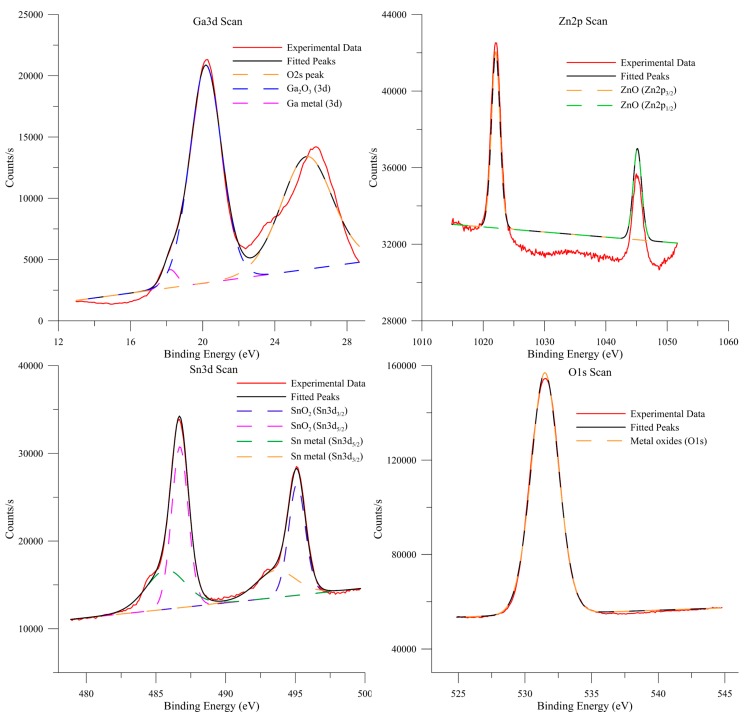
XPS spectra of Ga, Zn, Sn and O in oxide layers obtained from Ga–Sn–Zn eutectic alloy.

The chemical composition was analysed using X-ray photoelectron spectroscopy. The XPS spectra are presented in Figure 4. In the case of the gallium, the peak at 20.2 eV corresponds to Ga_2_O_3_, while the peak at around 25 eV is the O2s peak, which is in the same range as Ga. The peak observed at 18.2 eV is elemental Ga, which may indicate some inclusions of gallium in the obtained material. In the case of the zinc spectrum, the analysis is more complicated due to the fact that the difference between the binding energies of elemental zinc and zinc oxide is less than 1 eV. However, the main zinc peak is located at 1022 eV, which is closer to the ZnO oxide binding energy. In the case of tin, the peak around 485 eV can be identified as corresponding to metallic tin, while the peak at 486.7 eV corresponds to tin oxide SnO_2_.

It was determined that the chemical composition of the touch-printed layers is 88.1 atom % of gallium, 6.9 atom % of tin and 5.0 atom % of zinc. When oxygen is taken into account, the composition of the layer is as follows: 19.7 atom % of gallium, 1.6 atom % of tin, 1.1 atom % of zinc and 77.6 atom % of oxygen. It is crucial to note that, according to simple thermodynamics, the Ga_2_O_3_ (ΔG_f_ = −998.3 kJ/mol) phase is more likely to form than SnO_2_ or ZnO (ΔG_f_ = −515.8 and −350.5 kJ/mol, respectively) [23]. However, the presented results indicate the presence of not only gallium oxide but also tin and zinc oxides. It is possible that the tin and zinc substitute for gallium atoms in the Ga_2_O_3_ oxide lattice, or that some form of segregation occurs on the surface of the liquid alloy. It should also be taken into account that some tin and zinc oxide could be formed after the transfer occurs, and the metal left on the substrate before cleaning oxides.

**Figure 5:**
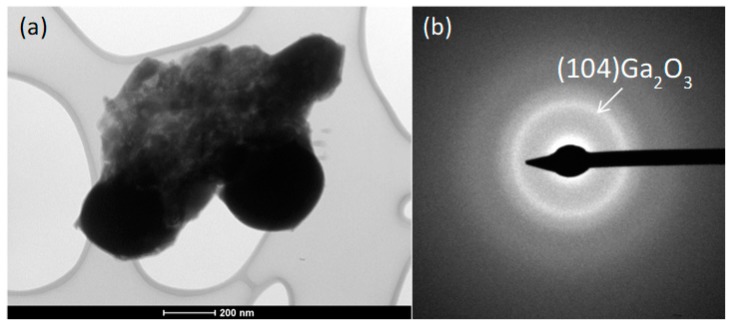
(**a**) TEM bright field image and (**b**) the corresponding selected area diffraction pattern.

**Figure 6:**
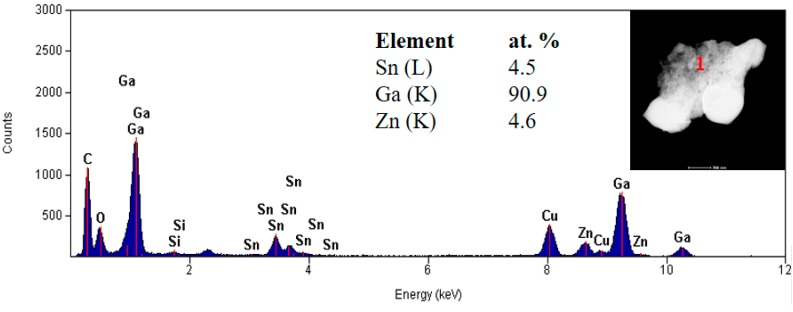
EDS point analysis of the chemical composition of the material. Insert: dark field image.

The TEM images are shown in Figure 5. The presented image shows a piece of the layer of approximately 1.2 × 0.8 μm. The oxide appears to be nanocrystalline. The material obtained on the TEM grid is much smaller than the material that can be obtained on non-porous surfaces, i.e., glass, silicon or quartz. Even though TEM grids with carbon film were used, the material does not transfer well onto the grids and does not appear in the form of clean sheets, but rather as folded pieces of oxide skin. EDS analysis was conducted, and the results are shown in Figure 6. 

Although the XPS and EDS results of the chemical composition investigation vary slightly, it can be concluded that they are consistent and indicate that the oxide layer is formed mainly of gallium oxide.

## 4. Conclusions

It is possible to obtain materials by transferring the oxide formed on liquid Ga–Sn–Zn alloy at room temperature onto different substrates, namely, quartz, glass and silicon. The contact angle between the liquid metal and the studied substrate (being in the range between 117° and 134°) does not affect the conducted measurements, although in this respect it could be interesting to study more hydrophobic and more hydrophilic surfaces. It is difficult to obtain macroscopic clear and smooth layers, due to folding of the oxide layer and oxidation of the metal on the analysed substrate. However, as can be seen from the atomic force image, it is possible to obtain smooth nanometric layers of a few micrometres in length. The XPS and EDS results suggest that the thin films obtained from eutectic Ga–Sn–Zn alloy contain not only gallium oxide, which should form according to thermodynamics, but also smaller quantities of tin and zinc oxides. The selective area diffraction pattern shows the presence of Ga_2_O_3_. It has not been determined whether the tin and zinc are substituting for gallium in the oxide or forming small patches of tin and zinc oxide in the main phase of gallium oxide. The study of liquid metal alloy surfaces should be undertaken as an important step in future research concerning alloys with low melting points, as it may prove crucial in many studied applications.
